# Staining Correction in Digital Pathology with Dye Amount Look-Up Table

**DOI:** 10.1155/2014/964708

**Published:** 2014-12-30

**Authors:** Pinky A. Bautista, Yukako Yagi

**Affiliations:** Department of Pathology, Massachusetts General Hospital, Boston, MA 02114, USA

## Abstract

Automated image analysis of histology images is affected by the staining variations in histology slides. In general, training images are used to optimize the parameters of an image analysis system. Color, being one of the dominant features of stained tissue samples, is being commonly utilized as feature to segment or classify the different stained tissue components. However, the colors impressed on the tissue components vary with the staining condition of the sample. Hence, when the staining conditions of the slides for the training and test images differ, the accuracy of the analysis results would likely degrade. In this work we present a method to correct the staining condition of the histology images by constructing a look-up table (LUT) of the stained pixels' dye amounts. The present method allows the user to not only correct the staining condition of a given histology image with respect to the staining condition of the reference slide, but to also recreate his/her preferred staining condition for the given image. The results of our experiments with hematoxylin and eosin (H&E) stained tissue images showed the effectiveness of the present method.

